# Regeneration of Different Plant Functional Types in a Masson Pine Forest Following Pine Wilt Disease

**DOI:** 10.1371/journal.pone.0036432

**Published:** 2012-05-01

**Authors:** Guang Hu, Xuehong Xu, Yuling Wang, Gao Lu, Kenneth J. Feeley, Mingjian Yu

**Affiliations:** 1 The Key Laboratory of Conservation Biology for Endangered Wildlife of the Ministry of Education, College of Life Sciences, Zhejiang University, Hangzhou, People's Republic of China; 2 Institute of Botany, Chinese Academy of Sciences, Beijing, People's Republic of China; 3 General Station of Forest Pest Management, State Forestry Administration, Shenyang, People's Republic of China; 4 Xiangshan Forest Pest Control and Quarantine Station, Xianshan, People's Republic of China; 5 Department of Biological Sciences, Florida International University, Miami, Florida, United States of America; Jyväskylä University, Finland

## Abstract

Pine wilt disease is a severe threat to the native pine forests in East Asia. Understanding the natural regeneration of the forests disturbed by pine wilt disease is thus critical for the conservation of biodiversity in this realm. We studied the dynamics of composition and structure within different plant functional types (PFTs) in Masson pine forests affected by pine wilt disease (PWD). Based on plant traits, all species were assigned to four PFTs: evergreen woody species (PFT1), deciduous woody species (PFT2), herbs (PFT3), and ferns (PFT4). We analyzed the changes in these PFTs during the initial disturbance period and during post-disturbance regeneration. The species richness, abundance and basal area, as well as life-stage structure of the PFTs changed differently after pine wilt disease. The direction of plant community regeneration depended on the differential response of the PFTs. PFT1, which has a higher tolerance to disturbances, became dominant during the post-disturbance regeneration, and a young evergreen-broad-leaved forest developed quickly after PWD. Results also indicated that the impacts of PWD were dampened by the feedbacks between PFTs and the microclimate, in which PFT4 played an important ecological role. In conclusion, we propose management at the functional type level instead of at the population level as a promising approach in ecological restoration and biodiversity conservation.

## Introduction

Pine wilt disease (PWD), caused by the invasive pine wood nematode (PWN, *Bursaphelencus xylophilus* Nickle), which is endemic to North America, is a severe threat to native pine forests and has also been spreading rapidly to other regions throughout the world [1], [2], [3], [4]. Outside North America, PWD was firstly found in Japan in 1905, in mainland China in 1982, in Taiwan in 1985, and in Korea in 1988 [1], [4]. In 1990s, PWD was also found in Nigeria and Portugal [3], [5]. In Mainland China, the invasion of the PWN was first recorded in 1982, and PWD has since been aggressively spreading to 186 counties and 15 provinces, infecting over one million hectares of pine forests from 1982 to 2011 [6], [7]. Despite the fact that the Chinese government is investing large amounts of money and effort to control the spread of PWD through the removal of infected and dead pine trees from damaged areas, and inspection and quarantine of pine timber before transportation, few effective methods of control have been found [6]. Given the rapid spread of PWD and the severe damage that it can cause, it is critical that we study the responses of the plant species in these infested forests to help develop effective management strategies.

Japanese red pine (*Pinus densiflora* Sieb. et Zucc.), Japanese black pine (*Pinus thunbergii* Parl.) and Masson pine (*Pinus massoniana* Lamb.) are the main hosts of PWN in East Asia. In these pine forests, PWN can be quickly transmitted from the infected pine to the healthy pines by pine sawyer beetles (*Monochamuns alternatus* Hope), and the leaves of pine trees will quickly wilt once infected by PWN [6]. Different from other natural and anthropogenic disturbances, PWD is a highly taxon-specific disease, infecting only pine trees [3], [8]. As such, most studies have focused on the responses of infected pine trees [3], [9], [10]. However, because the infected pine is often the dominant species where it occurs, PWD can also lead indirectly to different responses of other plant species during post-disturbance regeneration [2]. A few studies on the regeneration of forest communities following PWD infestation have been conducted in Japan [9], [11], [12] and China [5], [6], [13], indicating that PWD accelerated the rate of succession and shifted the dominant species in the forests. However, because of the complexity of the responses of individual species with different functional traits and life spans, these studies mostly referred to just a few dominant woody species in canopy or understory layers [2], [11], [14], [15], and the results cannot be applied to a broader understanding of the ecological response of all plants including herbaceous species.

Recent studies have begun to use plant functional types (PFTs) in describing the post-disturbance regeneration of natural ecosystems [16], [17]. In this context, PFTs are defined as groups of plant species which all respond similarly to environmental conditions and/or have similar functions in the communities and ecosystem processes [18], [19]. The concept of PFTs has been useful in predicting the responses of species to disturbances, describing successional processes, and guiding management practices [20], [21], [22], [23]. Combinations of plant functional traits have also been used to explain the variation in the spatial distribution of plant [24], resistance to disturbance [20], [25], [26], the dynamics of land use [18], and ecosystem services [27].

Based on our previous analyses of community structure and species diversity, we demonstrated that the PWN infection accelerated the succession from native pine plantations to zonal evergreen broad-leaved forests [6]. However, the underlying mechanisms driving the rapid conversion still require more exploration, and assessing the performance of PFTs in the community is likely to be a promising approach. In this study we address the specific questions of: (1) How do different PFTs respond to PWD infestation; (2) How does community structure shift within different life stages in the disturbance and post-disturbance regeneration stages; and (3) Are there compensation effects by the PFTs to the damage of PWD to the plant community during post-disturbance regeneration?

## Materials and Methods

### Study sites

**Figure 1:**
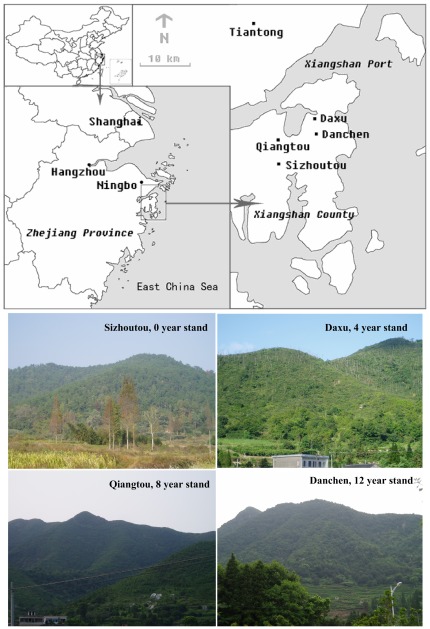
Map and photos of the study sites in Xianshan County, Zhejiang Province, East China. Sizhoutou: exposure to PWD 0 year stand, Daxu: 4 year stand, Qiangtou: 8 year stand, and Danchen: 12 year stand.

Study sites (29°14′–29°38′N, 121°32′–121°12′E) were located in Xiangshan, a coastal county close to the East China Sea (Fig. 1). This region has a subtropical monsoon humid climate. Mean annual temperature is approximately 16.5°C, with temperatures ranging from a minimum of −7.5°C to a maximum of 38.8°C (the mean temperature in January is 5°C and the mean temperature in July is 27°C). Total annual precipitation averages 1450 mm. Approximately 72% of forests (420 km^2^) in Xiangshan county are dominated by Masson pine [28]. Presently the county is considered to be one of the regions most severely damaged by PWD in China. Using a general forestry survey conducted by local governments, which provided the general forest characteristics and stand structure before and after PWN infestation, we selected four forests to study in Xiangshan with different histories of PWD infestation. The selected study forests (Sizhoutou, Daxu, Qiangtou, and Danchen) were all Masson pine plantations of similar density (3300–4500 stems/ha at planting), of similar ages (32–35 years), and with similar flora and habitat conditions (Table 1) prior PWD infestation. The forests have been infected with PWD for different amounts of time (Sizhoutou = 0 years - i.e., the forest was infected just prior to the study and did not yet exhibit significant damage to pine trees; Daxu = 4 years; Qiangtou = 8 years; and Danchen = 12 years) which allowed us to compare the impact of PWD on the structure and dynamics of the communities along a space-for-time chronosequence.

**Table 1:** Characteristics of the four study sites in Xianshan County, Zhejiang Province, East China.

Site	Years after infection	Elevation (m)	Slope (°)	Soil pH
Sizhoutou	0	43–126	24.1±8.70	4.46–4.80
Daxu	4	93–185	33.1±6.76	4.55–4.91
Qiangtou	8	79–153	29.1±3.87	4.82–5.26
Danchen	12	121–236	29.6±7.39	4.46–4.53

All sites are south-facing. Slope is the mean ± S. D. (N = 16) in each site. Soil pH is the range of humus layer, leached layer and illuvial layer.

### Data collection

Field surveys of the four study forests were conducted from August to October, 2003. Sixteen quadrats (10×10 m) were established in each forest at different topographic positions ranging from lower to upper hillside. All quadrats were separated by a minimum distance of 20 m. In each quadrat, all individual plants were identified to species [29] and measured for height and abundance. Individuals more than 1.3 m in height were also measured for their basal areas (cross sectional area at 1.3 m height). In order to classify plant species into PFTs (see below) we collected information on the suite of species traits including growth form (dependent on shoot architecture), life form (dependent on the position of buds), light-tolerance, leaf size, leaf phenology (the season of leaf shedding), leaf texture (the thickness and toughness of leaves), and leaf margin (Table 2). These functional traits were selected due to their linkage with resource utilization and tolerance to disturbance [30], [31], [32], [33]. Species traits were based on published species descriptions in Floras [29] and from field work according to the Cornelissen's handbook [33]. Twenty-four monitoring spots were randomly selected at each site for measuring light intensity under the tree canopy (3 m above the ground), under shrubs (1.2 m above the ground) and under the herb layer (0.1 m above the ground) with four replications between 11:00–14:00 local time in August (excluding cloudy and rainy days). The light intensity was also simultaneously measured in the open.

**Table 2:** Seven plant traits recorded for 160 species in the four study sites.

Plant Trait	Type	Description of classes in species-traits matrix
Growth form^b^	cat	tree = 1; shrub = 2; grass = 3; forb = 4; fern = 5
Life form^b^	cat	phanerophyte = 1; chamaephyte = 2; hemicryptophyte = 3; geophyte = 4; therophyte = 5
Light tolerance^a^^, ^ b	cat	shade-tolerant = 1; light-demanding = 2
Leaf size (cm^2^)^a^^, ^ b	cont	0–20 = 1; 20–100 = 2; 100–500 = 3; >500 = 4
Leaf phenology^a^^, ^ b	bin	evergreen = 1; summer-green = 2
Leaf texture^b^	cat	fimly = 1; orthophyll = 2; sclerophyll = 3
Leaf margin^b^	bin	entire = 1; non-entire = 2

Types of data were originally categorical (cat), continuous (cont) or binary (bin), and all were transformed to categorical data for cluster analysis.

a Based on Flora [29].

b Based on field observation.

### Data analysis

We used TWINSPAN cluster analysis to classify all plant species into 4 functional groups (PFT1, PFT2, PFT3, PFT4) based on their seven functional traits, and tested the results by Detrended Correspondence Analysis (DCA) [27]. Species richness, abundance, total basal area, and mean basal area were used to describe the PFTs within and between sites. Jaccard's similarity coefficients (ISJ) were used to evaluate the variation of species composition at each site. The individuals of woody plant species were divided into three life stages with different heights: (1) tree (≥3 m); (2) sapling (1∼3 m); and (3) seedling (<1 m). We calculated the frequency of each group in each quadrat.

We calculated relative light intensity (%) at each layer of each site using the values gathered from the different forest layers.

Two-way ANOVAs were used to compare the metrics of community structure (richness, abundance, total basal area and mean basal area) across the different PFTs and different life stages after PWD. Furthermore, Tukey's HSD test and paired sample T-tests were applied for multiple comparisons among all pairs of means. Pearson correlation analyses were used to test the correlation between light intensity and the abundances of PFTs. All statistical analyses were conducted in SPSS 16.0.

## Results

### PFTs classification

**Figure 2:**
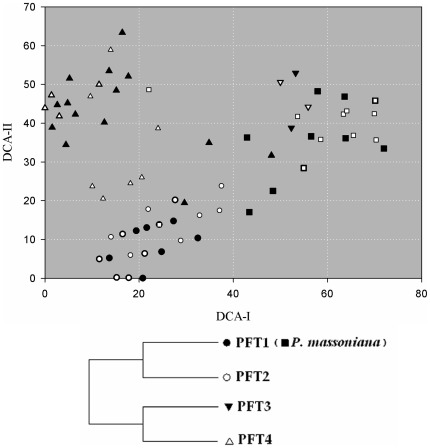
Detrended Correspondence Analysis (DCA) of the species×traits matrix. Plant functional types (top) defined by TWINSPAN (bottom) are described in Table 3. Masson pine (*P. massoniana*, dark square) is close to PFT1 (dark circle) indicating they have the similar traits.

We divided the 160 recorded species (Table S1) into four PFTs on the basis of TWINSPAN classification. The association among different PFTs (Fig. 2, top) was also testified by the distribution of PFTs in DCA analysis (Fig. 2, bottom). The main trend of variation reflected by DCA-axis I separated the species as being woody (PFTs 1 and 2) vs. non-woody (PFTs 3 and 4), and axis II mainly separated the woody species as being evergreen (PFT1) vs. summer-green species (PFT2). Further descriptions of the four PFTs are provided in Table 3.

**Table 3:** Summary of characteristics of four plant functional types (PFTs) clustered by TWINSPAN on a set of 160 species (species richness of each PFT shown in parentheses).

PFTs	Growth Form	Vegetative traits description^a^	Dominant species
PFT1(68)	Evergreen woody species	Tree or shrub; small thick evergreen leaf; high specific leaf area (SLA); long leaf lifespan; high tolerance to disturbance	*Pinus massoniana*; *Schima superba*; *Castanopsis sclerophylla*; *Lithocarpus glaber*; *Loropetalum chinense*
PFT2(55)	Deciduous woody species	Tree or shrub; large thin summer-green leaf; mostly light-demanding; low SLA; short leaf lifespan; fast growth rate	*Quercus fabri*; *Dalbergia hupeana*; *Liquidambar formosana*; *Albizia kalkora*
PFT3(26)	Grass, sedge and forb	Annual, biennial, or perennial; diversified types of leaves	*Lophatherum gracile*; *Carex breviculmis*; *Liriope spicata*; *Miscanthus sinensis*
PFT4(11)	Fern	Large thin leaf; light-demanding	*Dicranopteris dichotoma*; *Woodwardia japonica*

a Based on Flora and other references [29], [33].

### Responses of Woody PFTs (PFT1 & PFT2)

**Figure 3:**
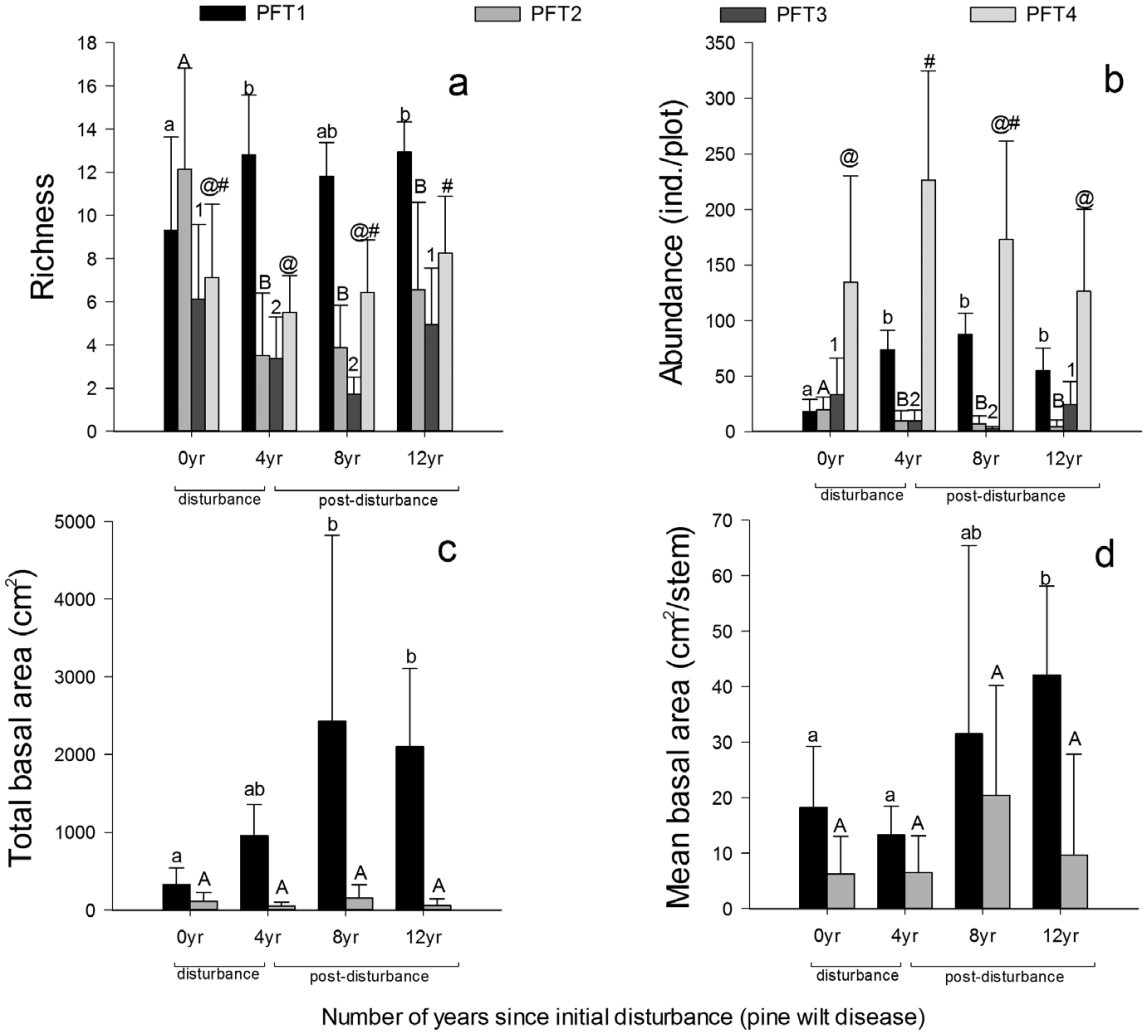
The different responses in dynamics of plant functional types. (a) richness, (b) abundance, (c) total basal area and (d) mean basal area during initial disturbance period and post-disturbance regeneration. Basal area data only refer to PFT1 and PFT2 and excludes individuals lower than 1.3 m height. Different labels above columns showed the significantly different means among different periods.

Significant interaction effects of PFTs and successional stages on four metrics of community structure indicated that the responses of the PFTs to PWD were markedly different at two different stages of forest recovery (Table 4), the initial disturbance period (0 to 4 years after PWD) and the post-disturbance regeneration (4 to 12 years). PFT2 had higher richness in the pine forest than PFT1 (paired T-test, t = −4.348, *p*<0.001) in the initial infection stage (i.e., in the forests of which had just been infected), but had the highest decrease in number of species during the period of initial disturbance (0 vs. 4 years post-infection, Fig. 3a). In the later post-disturbance period (4 to 12 years post-infection) species richness of PFT1 did not change significantly (Tukey's test, all *p*>0.05). In the post-disturbance period (4–12 yr), PFT2's richness remained lower than PFT1. Jaccard's similarity coefficients (ISJs) of PFT2 were also lower than those of PFT1 throughout the entire successional chronosequence (Table 5).

**Table 4:** Two-way ANOVAs of the effects of PFTs, successional stages after PWD, and their interaction on the four metrics of plant community structure.

Source	Sum of Squares	df	Mean Squares	F	*p*-value
**Richness (R^2^ = 0.63)**					
Intercept	1.30*10^4^	1	1.30*10^4^	1.56*10^3^	<0.001
Stage	4.31*10^2^	3	1.44*10^2^	17.24	<0.001
PFT	2.23*10^3^	3	7.43*10^2^	89.13	<0.001
Stage*PFT	8.11*10^2^	9	90.13	10.82	<0.001
Error	2.00*10^3^	240	8.33		
Total	1.85*10^4^	256			
**Abundance (R^2^ = 0.68)**					
Intercept	1.95*10^6^	1	1.95*10^6^	7.61*10^2^	<0.001
Stage	1.16*10^4^	3	3.87*10^3^	1.51	0.212
PFT	1.14*10^6^	3	3.81*10^5^	1.49*10^2^	<0.001
Stage*PFT	1.74*10^5^	9	1.94*10^4^	7.57	<0.001
Error	6.15*10^5^	240	2.56*10^3^		
Total	3.89*10^6^	256			
**Total basal area (R^2^ = 0.50)**					
Intercept	7.50*10^7^	1	7.50*10^7^	85.73	<0.001
Stage	2.33*10^7^	3	7.75*10^6^	8.86	<0.001
PFT	6.01*10^7^	1	6.01*10^7^	68.69	<0.001
Stage*PFT	2.32*10^7^	3	7.73*10^6^	8.84	<0.001
Error	1.05*10^8^	120	8.75*10^5^		
Total	2.87*10^8^	128			
**Mean basal area (R^2^ = 0.36)**					
Intercept	3.92*10^4^	1	3.92*10^4^	137.04	<0.001
Stage	6.16*10^3^	3	2.05*10^3^	7.18	<0.001
PFT	9.80*10^3^	1	9.80*10^3^	34.28	<0.001
Stage*PFT	3.39*10^3^	3	1.13*10^3^	3.96	0.010
Error	3.43*10^4^	120	2.86*10^2^		
Total	9.28*10^4^	128			

Basal area data only refer to PFT1 and PFT2 and excludes individuals lower than 1.3 m height.

**Table 5:** Jaccard's similarity coefficients (ISJ) of plant functional types between different regeneration stages after pine wilt disease indicated the species turnover rates of PFTs.

	0 yr	4 yr	8 yr
**PFT1**			
4 yr	0.465		
8 yr	0.488	0.625	
12 yr	0.463	0.5	0.531
**PFT2**			
4 yr	0.289		
8 yr	0.213	0.476	
12 yr	0.34	0.518	0.428
**PFT3**			
4 yr	0.158		
8 yr	0.21	0.5	
12 yr	0.036	0.364	0.231
**PFT4**			
4 yr	0.625		
8 yr	0.625	0.714	
12 yr	0.545	0.454	0.600

ISJ = *c*/(*a*+*b*+*c*), where *c* is the number of same species between different sites, *a* is the number of species only in one site, and *b* is the number of species only in another site.

The abundance and total basal area of PFT1 were both significantly greater than PFT2 after disturbance (4–12 yr, Fig. 3b, c), while mean basal area was not significantly different between these two groups (Fig. 3d). Two-way ANOVA analyses showed that the abundances of PFTs were significantly different between the initial disturbance and post-disturbance regeneration period (Table 4). In contrast to the decline of abundance in PFT2, Tukey's test showed that the abundance of PFT1 during post-disturbance regeneration (4, 8 and 12 yr) was significantly higher than the abundance during the initial infection stage (0 yr, all *p*<0.01). Total basal area of PFT1 had a similar trend as the abundance (Fig. 3c), while that of PFT2 remained low throughout the entire succession. Mean basal area of PFT1 showed a stable trend in the initial disturbance period, and increased in the post-disturbance period (Fig. 3d). Tukey's test indicated that mean basal area of PFT1 was significantly larger at the later regeneration stage than at the disturbance stage (0 vs. 12 yr, *p* = 0.006).

**Figure 4:**
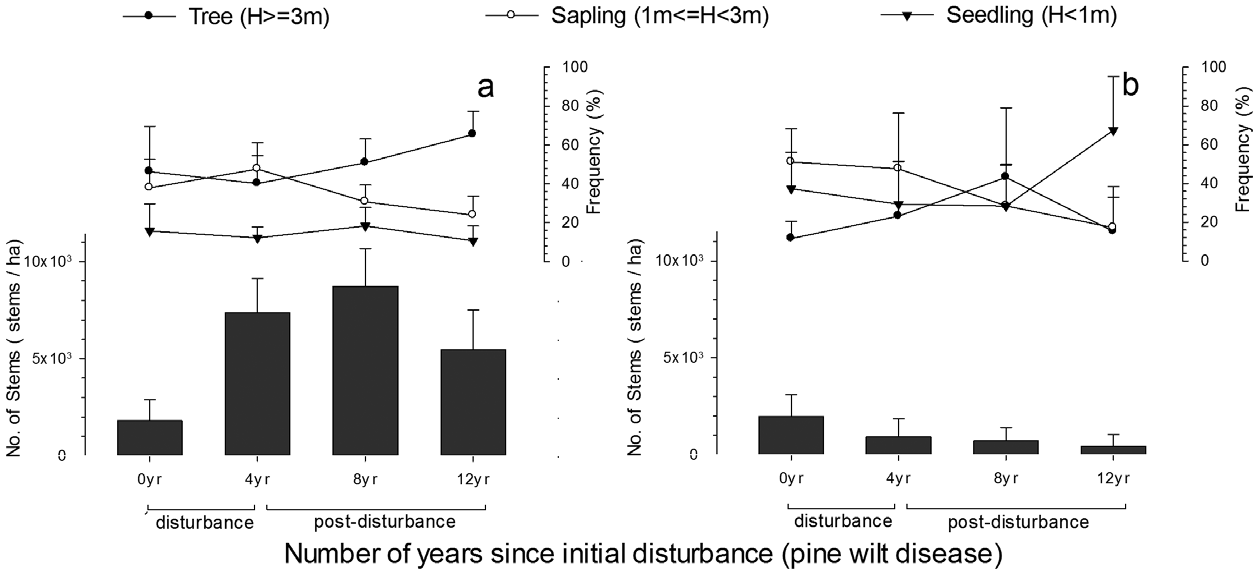
Stem density and life stage structure of PFT1 (a) and PFT2 (b) after pine wilt disease.

The different tree life stages also had different responses during the initial disturbance and post-disturbance regeneration periods (Fig. 4). The frequencies of tree groups had no significant changes in the initial disturbance period (0 vs. 4 yr, Tukey's test, PFT1, *p* = 0.714; PFT2, *p* = 0.369). Only the frequency of PFT1's tree group significantly increased at the later regeneration stage (0 vs. 12 yr, Tukey's test, *p* = 0.007). Paired T-tests showed that PFT1 had more tree individuals than saplings throughout the entire chronosequence (t = 3.76, *p*<0.001) while PFT2 had more sapling individuals (t = −3.92, *p*<0.001). The frequency of PFT1's saplings remained stable in the initial disturbance period (0 vs. 4 yr, Tukey's test, *p* = 0.112), then decreased during regeneration (0 vs. 12 yr, *p* = 0.008). The frequency of PFT2's saplings decreased during post-disturbance regeneration (0 vs. 8 and 12 yr, Tukey's test, both *p*<0.05). ANOVA showed that the frequency of PFT1's seedlings had no significant change during the entire process (F = 2.07, *p* = 0.113), while that of PFT2 increased in post-disturbance regeneration (0 vs. 12 yr, Tukey's test, *p* = 0.003).

### Responses of non-woody PFTs (PFT3 &PFT4)

Species richness in PFT3 decreased during the initial disturbance period (0 yr vs. 4 yr, Tukey's test, *p*<0.001). Despite the fact that PFT4 had a relatively stable species richness and higher ISJs in the comparison of species composition among different stages (Table 5), the abundance of PFT4 increased sharply in the initial disturbance period (0 yr vs. 4 yr, Tukey's test, *p* = 0.03), and then returned to initial levels (0 yr vs. 8 and 12 yr, both *p*>0.05). The abundance of PFT3 declined quickly following initial disturbance (0 yr vs. 4 yr, Tukey's test, *p* = 0.01), and returned in the later regeneration stage (0 yr vs. 12 yr, *p* = 0.06).

### Changes of light condition after PWD

**Figure 5:**
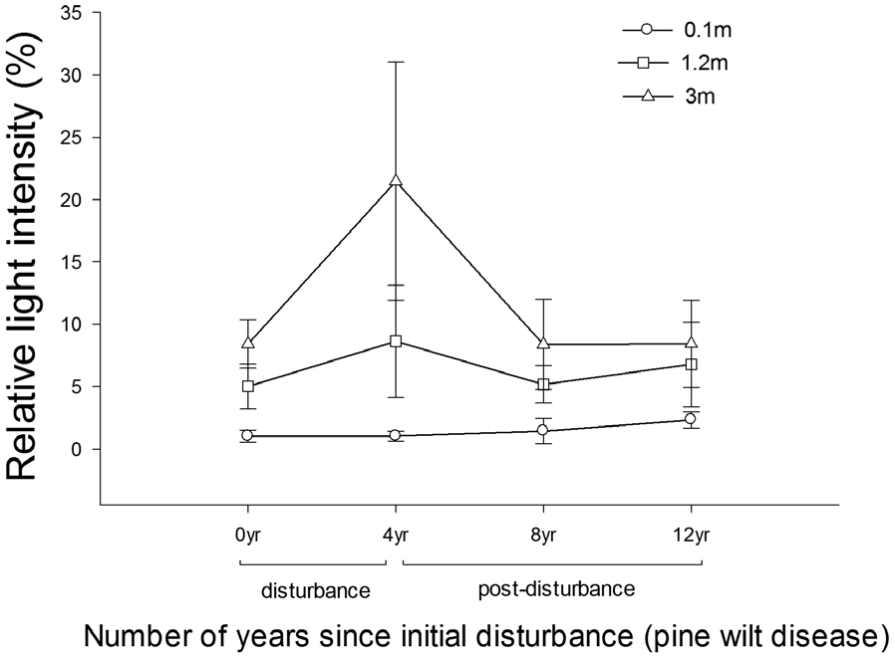
Relative light intensity at different layers in the forests affected by PWD.

The coverage of the higher canopy layer influenced the light intensity at the lower understory layers. Significant differences were found between the light intensity at 0.1 m, 1.2 m and 3 m heights (Fig. 5). The light intensity at 1.2 m and 3 m increased during the initial disturbance period, and then dropped to the pre-disturbance level during the later stages of the chronosequence. The light intensity near the ground (0.1 m) was negatively correlated with the abundance of PFT4 (*R*_0.1_ = −0.26, *P*<0.05). However, the light intensity at 1.2 m and 3 m was positively correlated with the abundance of PFT4 (*R*_3_ = 0.45, *P*<0.05; *R*_1.2_ = 0.26, *P*<0.05). There were no significant correlations between light intensity and abundance of other PFTs.

## Discussion

In this study, we used plant functional types (PFTs) for the first time to explore the responses of entire plant communities to disturbance by an invasive disease (PWD) and their ecological functions during the disturbance period and post-disturbance regeneration.

The loss of Masson pines due to PWD infection led immediately to an increase in richness and abundance of PFT1, but total basal area and mean basal area did not respond until the post-disturbance regeneration. The release of space and resources due to the loss of Masson pine enhanced the development of the suppressed PFT1 species. The growth of new seedlings and saplings caused the lag effects of basal area. For PFT2, the increase in abundance was consistent with the results from studies of Japanese forests which showed that the growths of PFT2 species were accelerated at the end of PWD due to their higher growth rates under strong light conditions [30], [31]. But the decrease of richness and total basal area indicated that this PFT2, especially the tree species, had weaker tolerance to disturbance. In contrast, the evergreen woody species of PFT1 had higher tolerance to the PWD disturbance [32], [33], [34]. The trade-off between tolerance and growth rate [35] determined the species composition and structure of the plant community after disturbance. In our study sites, the tolerance to the disturbance caused by PWD was more important for post-disturbance regeneration. Unstable species composition and higher species turnover as indicated by the lower ISJs of PFT2 also supported the importance of disturbance tolerance.

The different responses of PFTs drove the successional direction of regeneration after PWD. According to the increases of abundance, total basal area, and mean basal area, PFT1 will potentially become the dominant component in the canopy layer during regeneration. This pattern was not only due to the tolerance of PFT1, but also partly attributed to PFT1's increasing sapling abundance in the initial disturbance stage (0–4 yr, Fig. 4a) However, density-dependent self-thinning of the young trees decreased the total basal area of PFT1 during the later stages of regeneration [36], [37]. During post-disturbance regeneration, PFT2 was gradually suppressed by the development of PFT1 and re-closure of the canopy.

The life stage structure of different PFTs is also important to the pattern of secondary succession. The changes in frequency of the different life stages indicated different sensitivities of the PFT1 life stage groups to PWD in the initial disturbance period. The increased frequency in the tree group (Fig. 4a) made a large contribution to the abundance, total basal area and mean basal area of PFT1 (Fig. 3), which was due to the fast growth of saplings with the ecological release of space and resource after local extinction of some responsive species. The changes in frequency of PFT2 showed similar sensitivity as PFT1, but the tree groups decreased in regeneration due to the negative effects of PWD. It supported the importance of tolerance within the disturbed communities. The stable frequencies of PFT1 and PFT2 seedlings were due to the compensation effect of PFT4 as discussed below.

The change in microclimate after PWD was one main factor influencing the structure of the regenerating plant community. The light intensity and heterogeneity was increased after PWD at 1.2 m and 3 m height in the forests due to the loss of Masson pine overstory. Unexpectedly, we found that PFT4 (fern) increased rapidly after the PWD disturbance maintaining a stable micro-environment near the ground (Fig. 5). This effect has not been revealed in previous species-level studies. We speculate that the increased light condition at higher layers (1.2 m & 3 m) enhanced rapid growth of PFT4. The increasing abundance of PFT4 then had a negative effect on the light intensity near the ground (0.1 m), leading to a stable, low light intensity (Fig. 5). This indicates that there are feedbacks among the PFTs which may affect the microclimate of a site to mitigate the impact of disturbance [38], [39]. The sensitive and quick compensation growth of PFT4 after PWD provided a shady and “safe" environment (Fig. 5) for seed germination and seedling growth [40] of PFT1 but inhibited the shade-intolerant PFT2 in the disturbance period (0 yr–4 yr). At the later regeneration stage, the seedlings of PFT2 began to increase with the decline of PFT4 abundance due to canopy re-closure.

Knowledge on plant community responses during the process of regeneration following disturbances including human activities such as grazing, logging, and cultivating [20], [41], [42], and natural disasters such as typhoons/hurricanes, wildfires, and floods, etc. [12], [43], [44] can help in designing management strategies to effectively conserve biodiversity. Many large-scale disturbance events cause the indiscriminate destruction of most plant species forcing secondary succession to start from bare land [43], [45]. Consequently, pioneer species easily became dominant in the regenerating forests after these disturbances [46], [47], [48]. PWD, in contrast, is a taxon-specific disease only infecting pine trees. The responses of surviving plants suppressed by the dominant pine before the infection of PWD directly determined the direction of the post-PWD succession. Thus the succession pathways after PWD may vary depending on the forest age, floral composition, disturbance intensity, topography and human interference [2], [11], [49] which can affect the ecological patterns and processes in post-PWD regeneration. In this study, we used a PFT-level approach and found that a young evergreen-broad-leaved forest was quickly established following PWD. The tolerance of species to disturbance was more important than the growth rate in driving the direction of post-disturbance regeneration. Meanwhile, the responses of different life stages influenced the abundance and basal area of the woody PFTs. Furthermore, we found that the ecological compensation of PFT4 weakened the impact of PWD on seedling groups of woody PFTs and accelerated the shift from pine forest to evergreen forest after PWD. Based on our study, we suggest that management practices should focus at the functional type level rather than at the species level. In addition, minor components of the forests, such as herbs and ferns, may in fact have important ecological functions in the disturbance ecosystem and the post-disturbance regeneration. This will need more consideration in ecological restoration and biodiversity conservation.

## Supporting Information

Table S1 Plant species list of four study sites.
(DOCX)
