# Supplementary material for: Regeneration of Different Plant Functional Types in a Masson Pine Forest Following Pine Wilt Disease
Source: PLoS One. 2012 May 1;7(5):e36432. doi: 10.1371/journal.pone.0036432 (PMC3341345; doi:10.1371/journal.pone.0036432)
Supplement: Table S1 — Plant species list of four study sites. (DOCX) [file pone.0036432.s001.docx]

Table S1 Plant species list of four study sites. Species are listed by alphabetical order of family name. The detail description of the class of traits is shown in Table 2. GF: growth form (tree=1, shrub=2, grass=3, forb=4, fern=5); LF: life form (phanerophyte=1, chamaephyte=2, hemicryptophyte=3, geophyte=4, therophyte=5); LiT: light tolerance (shade-tolerant=1; light-demanding=2); LS: leaf size (0-20=1; 20-100=2; 100-500=3; >500=4); LP: leaf phenology (evergreen=1; summer-green=2); LT: leaf texture (fimly=1; orthophyll=2; sclerophyll=3); LM: Leaf margin (entire=1; non-entire＝2).

| Species | Family | GF | LF | LiT | LS | LP | LT | LM |
| --- | --- | --- | --- | --- | --- | --- | --- | --- |
| *Acer cordatum* | Aceraceae | 1 | 1 | 1 | 2 | 1 | 1 | 2 |
| *Rhus chinensis* | Anacardiaceae | 1 | 1 | 2 | 3 | 2 | 1 | 2 |
| *Toxicodendron succedaneum* | Anacardiaceae | 1 | 1 | 1 | 3 | 2 | 1 | 1 |
| *Ilex chinensis* | Aquifoliaceae | 1 | 1 | 1 | 2 | 1 | 3 | 2 |
| *Ilex ficoidea* | Aquifoliaceae | 1 | 1 | 1 | 1 | 1 | 3 | 1 |
| *Ilex rotunda* | Aquifoliaceae | 1 | 1 | 1 | 2 | 1 | 3 | 2 |
| *Aralia chinensis* | Araliaceae | 1 | 1 | 1 | 4 | 2 | 1 | 2 |
| *Aralia spinifolia* | Araliaceae | 2 | 1 | 1 | 4 | 2 | 1 | 2 |
| *Kalopanax septemlobus* | Araliaceae | 1 | 1 | 2 | 3 | 2 | 1 | 2 |
| *Asarum forbesii* | Aristolochiaceae | 4 | 4 | 1 | 2 | 2 | 2 | 1 |
| *Asplenium trichomanes* | Aspleniaceae | 5 | 3 | 1 | 3 | 2 | 3 | 2 |
| *Ainsliaea fragrans* | Asteraceae | 4 | 3 | 1 | 2 | 2 | 2 | 1 |
| *Ainsliaea macroclinidioides* | Asteraceae | 4 | 3 | 1 | 2 | 2 | 2 | 2 |
| *Artemisia anomala* | Asteraceae | 4 | 3 | 1 | 2 | 2 | 2 | 2 |
| *Aster ageratoides* | Asteraceae | 4 | 3 | 1 | 2 | 2 | 2 | 2 |
| *Aster turbinatus* | Asteraceae | 4 | 3 | 1 | 2 | 2 | 2 | 2 |
| *Conyza canadensis* | Asteraceae | 4 | 5 | 2 | 1 | 2 | 2 | 2 |
| *Eupatorium chinense* | Asteraceae | 4 | 3 | 1 | 2 | 2 | 2 | 2 |
| *Mahonia bealei* | Berberidaceae | 2 | 1 | 1 | 3 | 1 | 3 | 2 |
| *Carpinus londoniana* | Betulaceae | 1 | 1 | 1 | 2 | 2 | 1 | 2 |
| *Carpinus viminea* | Betulaceae | 1 | 1 | 1 | 2 | 2 | 1 | 2 |
| *Woodwardia japonica* | Blechnaceae | 5 | 3 | 1 | 3 | 2 | 3 | 2 |
| *Viburnum dilatatum* | Caprifoliaceae | 2 | 1 | 1 | 2 | 2 | 1 | 2 |
| *Viburnum erosum* | Caprifoliaceae | 2 | 1 | 1 | 2 | 2 | 1 | 2 |
| *Chloranthus spicatus* | Chloranthaceae | 2 | 2 | 1 | 2 | 2 | 2 | 2 |
| *Alangium chinense* | Cornaceae | 1 | 1 | 2 | 3 | 2 | 1 | 2 |
| *Macrocarpium officinale* | Cornaceae | 1 | 1 | 1 | 2 | 2 | 1 | 1 |
| *Cunninghamia lanceolata* | Cupressaceae | 1 | 1 | 2 | 1 | 1 | 3 | 1 |
| *Carex breviculmis* | Cyperaceae | 3 | 4 | 1 | 1 | 2 | 2 | 1 |
| *Carex hirticaulis* | Cyperaceae | 3 | 4 | 1 | 1 | 2 | 2 | 1 |
| *Daphniphyllum oldhamii* | Daphniphyllaceae | 1 | 1 | 1 | 2 | 1 | 3 | 1 |
| *Arachniodes pseudo-aristata* | Dryopteridaceae | 5 | 3 | 1 | 3 | 2 | 2 | 2 |
| *Cyrtomium fortunei* | Dryopteridaceae | 5 | 3 | 1 | 3 | 2 | 3 | 2 |
| *Dryopteris championii* | Dryopteridaceae | 5 | 3 | 1 | 3 | 2 | 2 | 2 |
| *Diospyros kaki* | Ebenaceae | 1 | 1 | 1 | 2 | 2 | 1 | 1 |
| *Diospyros morrisiana* | Ebenaceae | 1 | 1 | 1 | 2 | 1 | 3 | 1 |
| *Diospyros oleifera* | Ebenaceae | 1 | 1 | 1 | 2 | 2 | 1 | 1 |
| *Elaeagnus pungens* | Elaeagnaceae | 2 | 1 | 2 | 2 | 1 | 3 | 1 |
| *Rhododendron mariesii* | Ericaceae | 1 | 1 | 2 | 1 | 2 | 1 | 2 |
| *Rhododendron ovatum* | Ericaceae | 1 | 1 | 1 | 1 | 1 | 3 | 1 |
| *Rhododendron simsii* | Ericaceae | 2 | 1 | 2 | 1 | 2 | 1 | 2 |
| *Vaccinium bracteatum* | Ericaceae | 1 | 1 | 1 | 1 | 1 | 3 | 2 |
| *Vaccinium mandarinorum* | Ericaceae | 1 | 1 | 1 | 1 | 1 | 3 | 2 |
| *Glochidion puberum* | Euphorbiaceae | 1 | 1 | 2 | 1 | 2 | 1 | 1 |
| *Glochidion Wilsonii* | Euphorbiaceae | 2 | 1 | 1 | 1 | 2 | 1 | 1 |
| *Sapium seibiferum* | Euphorbiaceae | 1 | 1 | 2 | 2 | 2 | 1 | 1 |
| *Vernicia fordii* | Euphorbiaceae | 1 | 1 | 1 | 3 | 2 | 1 | 1 |
| *Vernicia montana* | Euphorbiaceae | 1 | 1 | 1 | 3 | 2 | 1 | 2 |
| *Albizia julibrissin* | Fabaceae | 1 | 1 | 2 | 3 | 2 | 1 | 1 |
| *Albizia kalkora* | Fabaceae | 1 | 1 | 2 | 3 | 2 | 1 | 1 |
| *Dalbergia hupeana* | Fabaceae | 1 | 1 | 1 | 2 | 2 | 1 | 1 |
| *Desmodium caudatum* | Fabaceae | 2 | 1 | 2 | 2 | 2 | 1 | 1 |
| *Hylodesmum podocarpium* | Fabaceae | 2 | 1 | 2 | 2 | 2 | 1 | 1 |
| *Indigofera decora* | Fabaceae | 2 | 1 | 1 | 2 | 2 | 1 | 1 |
| *Lespedeza formosa* | Fabaceae | 2 | 1 | 2 | 2 | 2 | 1 | 1 |
| *Ormosia henryi* | Fabaceae | 1 | 1 | 1 | 3 | 1 | 3 | 1 |
| *Castanea henryi* | Fagaceae | 1 | 1 | 1 | 2 | 2 | 1 | 2 |
| *Castanopsis carlesii* | Fagaceae | 1 | 1 | 1 | 1 | 1 | 3 | 2 |
| *Castanopsis fargesii* | Fagaceae | 1 | 1 | 1 | 2 | 1 | 3 | 1 |
| *Castanopsis sclerophylla* | Fagaceae | 1 | 1 | 1 | 2 | 1 | 3 | 2 |
| *Cyclobalanopsis gilva* | Fagaceae | 1 | 1 | 1 | 2 | 1 | 3 | 2 |
| *Cyclobalanopsis glauca* | Fagaceae | 1 | 1 | 1 | 2 | 1 | 3 | 2 |
| *Cyclobalanopsis gracilis* | Fagaceae | 1 | 1 | 1 | 2 | 1 | 3 | 2 |
| *Cyclobalanopsis mysinaefolia* | Fagaceae | 1 | 1 | 1 | 2 | 1 | 3 | 2 |
| *Cyclobalanopsis stewardiana* | Fagaceae | 1 | 1 | 1 | 2 | 1 | 3 | 2 |
| *Lithocarpus glaber* | Fagaceae | 1 | 1 | 1 | 2 | 1 | 3 | 1 |
| *Lithocarpus iteaphyllus* | Fagaceae | 1 | 1 | 1 | 2 | 1 | 3 | 1 |
| *Quercus acutissima* | Fagaceae | 1 | 1 | 2 | 2 | 2 | 1 | 2 |
| *Quercus fabri* | Fagaceae | 1 | 1 | 2 | 2 | 2 | 1 | 2 |
| *Dicranopteris dichotoma* | Gleicheniaceae | 5 | 3 | 2 | 3 | 2 | 2 | 2 |
| *Hicriopteris glauca* | Gleicheniaceae | 5 | 3 | 1 | 3 | 2 | 2 | 2 |
| *Liquidambar formosana* | Hamamelidaceae | 1 | 1 | 2 | 2 | 2 | 1 | 2 |
| *Loropetalum chinense* | Hamamelidaceae | 1 | 1 | 1 | 1 | 1 | 3 | 2 |
| *Platycarya strobilacea* | Juglandaceae | 1 | 1 | 2 | 3 | 2 | 1 | 2 |
| *Cinnamomum camphora* | Lauraceae | 1 | 1 | 1 | 2 | 1 | 3 | 1 |
| *Cinnamomum Chekiangense* | Lauraceae | 1 | 1 | 1 | 2 | 1 | 3 | 1 |
| *Lindera erythrocarpa* | Lauraceae | 1 | 1 | 1 | 2 | 2 | 1 | 1 |
| *Lindera glauca* | Lauraceae | 2 | 1 | 2 | 1 | 2 | 1 | 1 |
| *Lindera praecox* | Lauraceae | 1 | 1 | 1 | 1 | 2 | 1 | 1 |
| *Lindera rubronervia* | Lauraceae | 1 | 1 | 1 | 1 | 2 | 1 | 1 |
| *Litsea cubeba* | Lauraceae | 2 | 1 | 2 | 1 | 2 | 1 | 1 |
| *Litsea elongata* | Lauraceae | 1 | 1 | 1 | 2 | 1 | 3 | 1 |
| *Machilus pauhoi* | Lauraceae | 1 | 1 | 1 | 2 | 1 | 3 | 1 |
| *Machilus thunbergii* | Lauraceae | 1 | 1 | 1 | 2 | 1 | 3 | 1 |
| *Neolitsea aurata* | Lauraceae | 1 | 1 | 1 | 1 | 1 | 3 | 1 |
| *Liriope spicata* | Liliaceae | 4 | 4 | 1 | 1 | 2 | 2 | 2 |
| *Stenoloma chusanum* | Lindsaeaceae | 5 | 3 | 1 | 3 | 2 | 3 | 2 |
| *Ficus erecta* var. *beecheyana* | Moraceae | 1 | 1 | 1 | 2 | 2 | 1 | 2 |
| *Ficus formosana* | Moraceae | 1 | 1 | 1 | 1 | 2 | 1 | 1 |
| *Myrica rubra* | Myricaceae | 1 | 1 | 1 | 2 | 1 | 3 | 1 |
| *Ardisia crenata* | Myrsinaceae | 2 | 1 | 1 | 2 | 1 | 3 | 2 |
| *Ardisia crenata* | Myrsinaceae | 2 | 1 | 1 | 2 | 1 | 3 | 2 |
| *Ardisia japonica* | Myrsinaceae | 2 | 1 | 1 | 1 | 1 | 3 | 2 |
| *Syzygium buxifolium* | Myrtaceae | 1 | 1 | 1 | 1 | 1 | 3 | 1 |
| *Schoepfia jasminodora* | Olacaceae | 1 | 1 | 2 | 2 | 2 | 1 | 1 |
| *Fraxinus rhynchophylla* | Oleaceae | 1 | 1 | 1 | 2 | 2 | 3 | 2 |
| *Osmunda japonica* | Osmundaceae | 5 | 3 | 1 | 3 | 2 | 2 | 2 |
| *Pinus massoniana* | Pinaceae | 1 | 1 | 2 | 1 | 1 | 3 | 1 |
| *Arthraxon hispidus* | Poaceae | 3 | 5 | 1 | 1 | 2 | 2 | 1 |
| *Imperata koenigii* | Poaceae | 3 | 4 | 2 | 2 | 2 | 2 | 1 |
| *Indocalamus latifolius* | Poaceae | 3 | 4 | 2 | 2 | 2 | 2 | 1 |
| *Lophatherum gracile* | Poaceae | 3 | 3 | 1 | 1 | 2 | 2 | 1 |
| *Miscanthus floridulus* | Poaceae | 3 | 4 | 2 | 2 | 2 | 2 | 2 |
| *Miscanthus sinensis* | Poaceae | 3 | 4 | 2 | 2 | 2 | 2 | 2 |
| *Phyllostachys viridis* | Poaceae | 1 | 4 | 1 | 1 | 1 | 2 | 1 |
| *Pyrrosia lingua* | Polypodiaceae | 5 | 3 | 1 | 2 | 2 | 3 | 2 |
| *Lysimachia clethroides* | Primulaceae | 4 | 3 | 2 | 1 | 2 | 2 | 1 |
| *Berchemia kulingensis* | Rhamnaceae | 2 | 2 | 1 | 2 | 2 | 1 | 1 |
| *Rhamnus napalensis* | Rhamnaceae | 2 | 1 | 1 | 2 | 2 | 1 | 2 |
| *Laurocerasus spinulosa* | Rosaceae | 1 | 1 | 1 | 2 | 1 | 3 | 2 |
| *Photinia serrulata* | Rosaceae | 1 | 1 | 1 | 2 | 1 | 3 | 2 |
| *Potentilla chinensis* | Rosaceae | 4 | 2 | 2 | 2 | 2 | 2 | 2 |
| *Raphiolepis indica* | Rosaceae | 1 | 1 | 1 | 1 | 1 | 3 | 2 |
| *Rosa laevigata* | Rosaceae | 2 | 1 | 1 | 2 | 1 | 3 | 2 |
| *Rubus amphidasys* | Rosaceae | 2 | 2 | 1 | 2 | 1 | 3 | 2 |
| *Rubus buergeri* | Rosaceae | 2 | 2 | 1 | 2 | 1 | 3 | 2 |
| *Rubus corchorifolius* | Rosaceae | 2 | 1 | 2 | 1 | 2 | 2 | 2 |
| *Rubus coreanus* | Rosaceae | 2 | 1 | 2 | 2 | 2 | 2 | 2 |
| *Damnacanthus indicus* | Rubiaceae | 2 | 1 | 1 | 1 | 1 | 3 | 1 |
| *Gardenia jasminoides* | Rubiaceae | 2 | 1 | 1 | 2 | 1 | 3 | 1 |
| *Hedyotis chrysotricha* | Rubiaceae | 4 | 3 | 1 | 1 | 2 | 1 | 1 |
| *Lasianthus japonicus* | Rubiaceae | 2 | 1 | 1 | 2 | 1 | 1 | 1 |
| *Serissa japonica* | Rubiaceae | 2 | 1 | 2 | 1 | 2 | 1 | 1 |
| *Tarenna mollissima* | Rubiaceae | 1 | 1 | 1 | 2 | 2 | 1 | 1 |
| *Itea oblonga* | Saxifragaceae | 2 | 1 | 1 | 2 | 1 | 3 | 2 |
| *Siphonostegia laeta* | Scrophulariaceae | 4 | 5 | 1 | 1 | 2 | 2 | 2 |
| *Ailanthus altissima* | Simaroubaceae | 1 | 1 | 2 | 3 | 2 | 1 | 2 |
| *Euscaphis japonica* | Staphyleaceae | 1 | 1 | 2 | 3 | 2 | 1 | 2 |
| *Alniphyllum fortunei* | Styracaceae | 1 | 1 | 1 | 2 | 2 | 1 | 2 |
| *Styrax confusus* | Styracaceae | 1 | 1 | 1 | 2 | 2 | 1 | 2 |
| *Styrax faberi* | Styracaceae | 2 | 1 | 1 | 2 | 2 | 1 | 2 |
| *Styrax odoratissimus* | Styracaceae | 1 | 1 | 1 | 1 | 2 | 1 | 2 |
| *Symplocos anomala* | Symplocaceae | 1 | 1 | 1 | 1 | 1 | 3 | 2 |
| *Symplocos heishanensis* | Symplocaceae | 1 | 1 | 1 | 2 | 1 | 3 | 1 |
| *Symplocos lancifolia* | Symplocaceae | 1 | 1 | 1 | 1 | 1 | 3 | 2 |
| *Symplocos laurina* | Symplocaceae | 1 | 1 | 1 | 2 | 1 | 3 | 2 |
| *Symplocos paniculata* | Symplocaceae | 1 | 1 | 2 | 2 | 2 | 1 | 2 |
| *Symplocos setchuensis* | Symplocaceae | 1 | 1 | 1 | 2 | 1 | 3 | 2 |
| *Symplocos stellaris* | Symplocaceae | 1 | 1 | 1 | 2 | 1 | 3 | 1 |
| *Symplocos sumuntia* | Symplocaceae | 1 | 1 | 1 | 1 | 1 | 3 | 2 |
| *Camellia chekiang* | Theaceae | 1 | 1 | 1 | 2 | 1 | 3 | 2 |
| *Camellia cuspidata* | Theaceae | 2 | 1 | 1 | 1 | 1 | 3 | 2 |
| *Camellia fraterna* | Theaceae | 2 | 1 | 1 | 1 | 1 | 3 | 2 |
| *Camellia oleifera* | Theaceae | 1 | 1 | 1 | 2 | 1 | 3 | 1 |
| *Camellia sinensis* | Theaceae | 1 | 1 | 1 | 2 | 1 | 3 | 2 |
| *Eurya muricata* | Theaceae | 1 | 1 | 1 | 2 | 1 | 3 | 2 |
| *Eurya nitida* | Theaceae | 1 | 1 | 1 | 1 | 1 | 3 | 2 |
| *Eurya rubiginosa* | Theaceae | 1 | 1 | 1 | 2 | 1 | 3 | 2 |
| *Schima superba* | Theaceae | 1 | 1 | 1 | 2 | 1 | 3 | 2 |
| *Ternstroemia gymnanthera* | Theaceae | 1 | 1 | 1 | 2 | 1 | 3 | 1 |
| *Parathelypteris glanduligera* | Thelypteridaceae | 5 | 3 | 1 | 3 | 2 | 3 | 2 |
| *Tilia endochrysea* | Tiliaceae | 1 | 1 | 1 | 2 | 2 | 2 | 2 |
| *Gonostegia hirta* | Urticaceae | 4 | 3 | 1 | 1 | 2 | 2 | 1 |
| *Callicarpa cathayana* | Verbenaceae | 2 | 1 | 2 | 2 | 2 | 1 | 2 |
| *Callicarpa formosana* | Verbenaceae | 2 | 1 | 1 | 2 | 2 | 1 | 2 |
| *Callicarpa giraldii* | Verbenaceae | 2 | 1 | 2 | 2 | 2 | 1 | 2 |
| *Clerodendrum cyrtophyllum* | Verbenaceae | 1 | 1 | 1 | 2 | 2 | 1 | 2 |
| *Premna microphylla* | Verbenaceae | 2 | 1 | 2 | 2 | 2 | 1 | 2 |
| *Vitex negundo* | Verbenaceae | 1 | 1 | 2 | 2 | 2 | 1 | 2 |
| *Zingiber mioga* | Zingiberaceae | 4 | 4 | 1 | 2 | 2 | 2 | 1 |
